# Effects on Performance, Immunological Response and Short-Chain Fatty Acid Profile in Feces of Nursery Piglets Fed with Organic Acids and Yeast Wall

**DOI:** 10.3390/ani15071051

**Published:** 2025-04-04

**Authors:** Cassio Antônio Ficagna, Aleksandro Schafer da Silva, Rafael Domingos Rofino, Emerson Zatti, Tatiane Esposito, Ana Carolina H. Xavier, Roger Wagner, Bianca Fagan Bissacotti, Ronaldo Barbieri Seghetto, Eduardo Mioto Ternus, Diovani Paiano

**Affiliations:** 1 Programa de Pós-Graduação em Zootecnia, Universidade do Estado de Santa Catarina, Chapecó 89815-630, Brazil; cassioficagna98@gmail.com (C.A.F.); rafael.dar@edu.udesc.br (R.D.R.); emersonzatti@gmail.com (E.Z.); 2 Departamento de Zootecnia, Universidade do Estado de Santa Catarina, Chapecó 89815-630, Brazil; tatiane.esposito04@edu.udesc.br (T.E.); diovani.paiano@udesc.br (D.P.); 3 Departamento de Ciências de Alimento, Universidade Federal de Santa Maria, Santa Maria 97105-900, Brazil; anacarolinahadlich5@gmail.com (A.C.H.X.); rogerwag@gmail.com (R.W.); 4 Departamento de Zootecnia, Universidade Federal de Santa Catarina, Florianópolis 88040-900, Brazil; bianca_fbissacotti@hotmail.com; 5 Programa de Pós-Graduação em Bioquímica Toxicológica, Universidade Federal de Santa Maria, Santa Maria 97105-900, Brazil; ronaldobarbieriseghetto@edu.ufsc.br; 6 Programa de Pós-Graduação em Ciência Animal, Universidade do Estado de Santa Catarina, Lages 88520-000, Brazil; eduardo.ternus@edu.udesc.br

**Keywords:** nursery piglets, formic acid, acetic acid, mannan oligosaccharides, beta-glucans

## Abstract

The piglet nursery phase is one of the critical moments in production, especially in the first few weeks after weaning. Growth-promoting antibiotics have always been used in this phase, but the world is banning or limiting the use of antibiotics for this purpose, which has led researchers to seek alternatives, with an emphasis on organic and natural ones. As a result, this study aimed to evaluate whether a combination of organic acids and their derivatives (ammonium formate, formic acid, ammonium propionate, and acetic acid) and yeast wall (mannan oligosaccharides and beta-glucans) in piglet feeding during the nursery phase has positive effects on the SCFA profile in feces and animal health reflected in greater weight gain. A 40-day experiment was conducted in a completely randomized design containing three treatments, each with nine replicates and three piglets per replicate: negative control (NC: without additives), and a combination of organic acids and yeast wall at doses of 1 and 2 kg/ton (AO+YW-1 and AO+YW-2, respectively). Animals received four diets: pre-starter 1 (d1–7), pre-starter 2 (d8–14), starter 1 (d15–25), and starter 2 (d26–40). The animals were weighed on the day of the diet change. Blood and feces were collected on days 14 and 40 of the experiment. Piglets from the AO+YW-1 group showed more significant weight gain than NC. Feed intake was higher in piglets from the AO+YW-1 group considering the first 25 days of the experiment compared to NC; there was no treatment effect on feed conversion. Piglets from NC had higher levels of C-reactive protein and ferritin, while AO+YW-2 had higher levels of interleukin 10 and lower levels of TNF-α. A greater quantity of SCFA was observed in the feces of piglets on d40, related to the changes in propionic, butyric, isovaleric, and valeric acids. Therefore, the combination of organic acids and yeast wall used at a dose of 1 kg/ton proved to be an additive option for the diet of piglets in the nursery phase to enhance weight gain and reduce the number of doses of injectable antibiotics.

## Simple Summary

The combination of organic acids and yeast wall is a feed additive option for piglets in the nursery phase to enhance weight gain and reduce the number of doses of injectable antibiotics. The consumption of the additive stimulated the production of erythrocytes and had an anti-inflammatory effect, something positive in production animals. The ingestion of the combination of organic acids and prebiotics also changed the short-chain fatty acid (SCFA) in the feces of piglets. The combination of additives was beneficial to animal health and, consequently, to growth performance.

## 1. Introduction

The use of antibiotics as conventional growth promoters in animal production has always been the subject of discussions that associate this activity with the increase/selection in/of resistant bacterial strains in humans [1]. Given that this problem could involve public health, the central regulatory authorities have created restrictions involving this practice to force researchers and industry to seek new alternatives to minimize the withdrawal of these additives in pig farming. Thus, alternative additives are tested to minimize such problems and the effects of the withdrawal of antimicrobials [2]. Organic acids are an alternative, as they have antimicrobial action, promote the growth of intestinal cells, and improve feed conversion [3]. The most frequently used organic acids are short-chain fatty acids (SCFAs), such as formic acid, propionic acid, butyric acid, acetic acid, citric acid, and malic acid. These are weak organic acids, and, when dissolved in water, part is transformed into hydrogen and hydroxyl ions. Furthermore, these acids’ functioning and mode of action depend on their pH and pKa value [1] and can be provided to birds via water and/or feed. Organic acids such as acetic, formic, and propionic acids generally aim to reduce gastric pH, provide an antimicrobial effect in the digestive tract, and increase the action of digestive enzymes, especially pepsins, which improve protein digestibility. Furthermore, it is known that organic acids act on the physiology of the intestinal mucosa and maintain the integrity and height of the intestinal villi with a more significant number of viable cells [4]. It is also well known that using organic acids for weaned piglets improves animal performance, reducing antibiotic use and costs [5,6].

Another alternative to antibiotic replacement is prebiotics, generally carbohydrates that are not digestible in the upper part of the gastrointestinal tract (GIT) of animals, constituting a selective substrate for specific beneficial bacteria in the digestive tract [7]. Prebiotics are considered polymers that are not digestible for mammals (β-type bonds). Still, they can be fermented by the autochthonous microbiota and favorably alter the composition and activity of intestinal microorganisms [8]. This, in turn, increases the production of beneficial bacterial metabolites, such as SCFAs, and promotes the growth of beneficial bacteria [9]. Intestinal health-promoting bacteria can ferment prebiotics, improving intestinal microbial structures, the integrity of intestinal epithelial cells, and animal health [10]. Mannan oligosaccharides (MOS), yeast beta-glucan, and fructans are the main prebiotics widely studied and successfully applied in animal production [2,10]. MOS and beta-glucans are examples of prebiotics extracted from the cell wall of yeast that are intended to help maintain digestive efficiency and integrity of the intestinal epithelium, with increased nutrient absorption and positive stimulation of the animal’s immune system at the intestinal level [7,11].

It is believed that mixtures of feed additives are more effective in developing the digestive tract regarding the profile of microorganisms. In this line, it is known that the dietary addition of acidifying mixtures improves the growth performance of piglets by promoting the positive modulation of the intestinal microbiota associated with better protein digestibility, as already described in the literature [12]. Therefore, the hypothesis is that combining organic acids and prebiotics causes immunological stimulation and modulation of the SCFA profile in the GIT, reducing the number of sick animals that need to be medicated, consequently enhancing growth performance. Therefore, this study aimed to evaluate whether the combination of organic acids (formic acid and acetic acid) and yeast wall (MOS and beta-glucans) in piglets during the nursery phase has positive effects on growth, SCFA profile in feces, and animal health.

## 2. Materials and Methods

### 2.1. Additive

In this study, we used additive formulated with 25 g of yeast wall of *Saccharomyces cerevisiae* (85% mannan oligosaccharides and 15% beta-glucans) and organic acids and their derivatives (10.30 g of ammonium formate, 5.15 g of formic acid, 3.43 g of ammonium propionate, 0.86 g of acetic acid, 12 g of water and 100 g of vermiculite). The product is presented in a protected form inside vermiculite. It is essential to make it clear that this product is not intended to act as a growth promoter but rather as a functional and therapeutic additive, aiming at preventing diseases and, thus, favoring the development of piglets in the nursery phase, a critical and challenging time.

### 2.2. Animals and Facilities

The ethics committee approved this study for using animals in research at the Universidade do Estado de Santa Catarina, protocol number 2591240422. The experiment was conducted in the Experimental Farm of UDESC Oeste (FECEO) swine sector in Guatambu/SC. This study used 81 non-castrated piglets (weaned males aged 28 days; mean weight of 7.17 kg), commercial lineage originating from the crossing of sows (landrace x large white—Aurora^®^ genetics, Chapecó, Brazil) with a terminator male (AGPIC 337—Agroceres^®^ genetics, Paranavaí, Brazil). The animals were housed at the experimental station within 4 h after being separated from their mothers. Animals with similar body weights were selected (we used a portable scale for this purpose). We used apparently healthy animals, with offspring of sows subjected to the farm’s vaccination immunization protocol before parturition. The animals were transported by truck (transport time of 15 min) as soon as they arrived at the house in an experimental shed. Upon arrival, they were weighed, received earrings with different numbers, and were distributed evenly in the pens.

In the experimental room, the temperature was controlled by an automatic heater, programmed for each week of the piglet’s stay in the nursery. Likewise, the lighting in the shed was automatically controlled according to the age of the piglets, providing periods of darkness at night. The ventilation of the experimental area was carried out daily, using curtains. In the initial phase, the curtains were left open during the day, in order to ventilate the environment.

### 2.3. Experimental Design and Diets

Piglets were divided into three treatments, nine replicates per treatment and three animals per replicate; each pen was considered a replicate/experimental unit. The piglets were placed in pens (90 cm × 1.20 m) equipped with trough-type feeders and nipple drinkers (minimum flow rate of 1.5 L/min/pen). The experimental period was 40 days, subdivided into four phases, called pre-initial I (0–7 days), pre-initial II (8–15 days), initial I (15–28 days), and initial II (29–40 days). The piglets received ad libitum feed (powdered feed up to 1 mm), formulated for minimum cost according to the requirements and nutritional composition of the feeds established in the Brazilian Tables of Poultry and Swine [12]. All treatments received the same basal diet (Table 1), to which the following additives were added: NC—negative control, without antimicrobials and additives, and combination of organic acids and yeast wall at doses of 1 and 2 kg/ton (AO+YW-1 and AO+YW-2, respectively). It is important to make it clear that a dose of 1 kg/ton is the one recommended by the manufacturer; a dose of 2 kg/ton was chosen due to the history of high challenge present in the experimental environment (test dose). After production of the basal feed under commercial factory conditions, in the experimental sector, using a horizontal mixer, the organic acids and yeast cell walls were added according to each treatment, and 5 min was standardized for each mixture.

### 2.4. Data and Sample Collection

The piglets were weighed at the piglet house at the end of each period (days 7, 14, 25, and 40) on the same date as the feed change. Feed intake per pen was measured throughout the experiment, allowing the determination of the average daily consumption and the consumption per period of each feed. The feed was provided in eight daily moments, performed by the authors of this work daily, being calculated by period (d1–7; d8–14; d15–25; d26–40) based on the formula: Feed intake = provided − leftover.

The weight gain (WG) between the rearing phases and the experimental period was determined: WG = final weight − initial weight. With this information, the feed conversion (FCR) was calculated, and the total amount of feed ingested was divided by the WG in the determined consumption measurement period.

On days 14 and 40, blood samples were collected by puncturing the cranial vena cava using needles (25 × 7 mm) of one piglet per pen, using the piglets with the weight closest to the pen average as the criterion. The blood collected (2 mL) was placed in a tube with 10% EDTA for blood count analysis. Another tube (4 mL) without anticoagulant was centrifuged (10 min at 920× *g*), and the serum was extracted, placed in microtubes, and stored at −20 °C for later analysis.

Fresh piglet feces was also collected on the 14th and 40th days (one sample per pen immediately after the animal defecated). The sample was then placed in a plastic bag and the freezer (−20 °C) until analysis.

### 2.5. Laboratory Analyses

#### 2.5.1. Blood Count

Within three hours after collection, the Vet electronic device (model 3000, Equip, Itatiba, SP, Brazil) was used to analyze hemoglobin concentration, total number of erythrocytes and leukocytes, and hematocrit percentage. A blood smear was made and stained with a rapid Panotico kit for counting under an optical microscope at 1000× magnification for the leukocyte differential.

#### 2.5.2. Serum Biochemistry

Serum albumin and total protein levels were assessed using commercial kits on semi-automatic Bio Plus 2000^®^ equipment (Bioplus Produtos para Laboratórios Ltda, Barueri, SP, Brazil). Globulin levels were obtained by subtracting total protein from albumin.

#### 2.5.3. Cytokine Profile, C-Reactive Protein, and Ferritin

The cytokines TNF-α and interleukin-10 (IL-10) were evaluated by ELISA using commercial kits according to the manufacturer’s instructions (eBioscience, San Diego, CA, USA). According to the manufacturer’s instructions, C-reactive protein and ferritin were measured using commercial kits (Analisa^®^, Campinas, Brazil).

#### 2.5.4. Short-Chain Fatty Acid Profile in Feces

For SCFA analysis, 15 mL of distilled water was added to 3 g of feces in a test tube of 50 mL. The mixture was homogenized for 30 s in a vortex homogenizer and taken to the shaking table for 5 min. Then, the mixture was centrifuged at 630× *g* for 3 min, and 1 mL of the supernatant was centrifuged again in polypropylene microtubes (2 mL) for 5 min (12,300× *g*). After, an aliquot of 250 μL of sample supernatant was added to 250 μL of formic acid in polypropylene microtubes. It was shaken in a vortex and once more centrifuged for 5 min (12,300× *g*); 250 μL of the supernatant of the mixture was collected in another polypropylene tube previously containing an internal standard composed of isoamyl alcohol (640 µg mL^−1^ in methanol) and passed through the last centrifugation. Then, 600 μL of the sample was inserted into an injection vial, and 1 μL was injected (split mode at 1:10) into a gas chromatograph equipped with a flame ionization detector (GC-FID; Varian Star 3400, Palo Alto, CA, USA) and an autosampler (Varian 8200CX, Palo Alto, CA, USA). The carrier gas used was hydrogen at a constant pressure of 20 psi. The analytes (acetic, propionic, butyric, valeric, and isovaleric acids) were separated by a CP WAX 52CB capillary column (60 m × 0.25 mm; 0.25 μm stationary phase thickness). The initial column temperature was set at 80 °C for 1 min and increased to 120 °C at a rate of 8 °C min^−1^, then to 230 °C for 20 °C min^−1^, where it remained for 1 min. The injector and detector temperatures were set to 250 °C. Method validation comprised the following parameters: selectivity, linearity, linear range, repeatability, precision, limit of detection (LOD), and limit of quantification (LOQ) for acetic, propionic, butyric, and isovaleric acids (Table S1). Linearity was assessed by calculating a regression equation using the least squares method. LOD and LOQ values were obtained by sequential dilutions to signal-to-noise ratios of 3:1 and 6:1, respectively. Precision was assessed by analyzing the repeatability of six replicated samples. Accuracy was determined by recovering known amounts of standard substances added to a diluted sample. The valeric acid was expressed as the equivalent of isovaleric acid. The results were expressed in mmol of each SCFA per kg^−1^ of feces.

### 2.6. Statistical Analyses

The experimental data were first analyzed descriptively; measures of central tendency (median) and data dispersion (range that stands for the interval between the minimum and maximum values in the data) were computed. Further, all variables were subjected to the Shapiro–Wilk W-test, which revealed a normal data distribution. Skewness, kurtosis, and homogeneity were evaluated using the Levene test, and linearity was used for linear regression. All data were normally distributed and met the standard for parametric data analysis. All data were analyzed using the SAS ‘MIXED’ procedure (SAS Inst. Inc., Cary, NC, USA; version 9.4). Satterthwaite approximation was used to determine the denominator degrees of freedom for the fixed effects test. The variables WG, feed intake, and feed conversion were tested for the fixed effect of treatment, using pen (treatment) as a random effect. Body weight was analyzed as repeated measures and tested for fixed effects of treatment, day, and treatment × day interaction, using pen (treatment) as a random effect. Blood variables and fecal fatty acids were analyzed as repeated measures and tested for fixed effects of treatment, day, and treatment × day interaction, using animal (treatment) as a random effect. The treatment × day interaction allows us to identify whether the tested additive was effective during the experimental period or occurred at specific times, as well as whether there were fluctuations during this study. Body weight results from day 1 were included as an independent covariate. The first-order autoregressive covariance structure was selected according to the lowest Akaike information criterion. Means were separated by the PDIFF method (Tukey test for comparison of means between groups), and all results were reported as LSMEANS followed by SEM. Significance was defined when *p* ≤ 0.05 and trend when *p* > 0.05 and ≤0.10.

## 3. Results

### 3.1. Clinical Signs/Pathologies in Piglets

In the first week of the experiment, the piglets had health problems, resulting in generalized diarrhea and many animals coughing; these animals were medicated with linomycin (15 mg/kg BW, intramuscular) for 3 to 5 consecutive days at 24 h intervals, but in cases of encephalitis and persistent cough, a combination of colistin and amoxicillin was used (Agroplus^®^, Virbac Brasil, Sorocaba, SP, Brazil) (1 mL/10 kg, intramuscular per 3 to 5 days at 24 h intervals). The diagnosis of changes and diseases was made by a veterinarian, as well as the choice of drug and the duration of medication. The number of animals medicated was 14 piglets (NC group), 4 piglets (AO+YW-1 group), and 11 piglets (AO+YW-2 group), as detailed in Table 2.

### 3.2. Performance

Growth performance results were presented in Table 3. On day 40, there was a treatment × day interaction, with a higher body weight being observed in piglets from AO+YW-1 compared to NC (*p* = 0.05). Greater weight gain of piglets from AO+YW-1 compared to the other groups was observed between days 1 and 14 (*p* = 0.01), days 1 and 26 (*p* = 0.01) and days 1 and 40 (*p* = 0.01). Between days 1 and 26, a higher feed intake of piglets from the AO+YW-1 group was observed compared to the other two groups (*p* = 0.02), while, considering the period from 1 to 40 days, piglets from the AO+YW-2 group had the lowest feed intake when compared to AO+YW-1 and NC (*p* = 0.05). In the periods analyzed, only between days 1 and 14 was a difference observed between groups for feed conversion, being greater in piglets from AO+YW-2 when compared to AO+YW-1 and NC (*p* = 0.01).

### 3.3. Hematologic Parameters

Hematological results are presented in Table 4. There was a treatment × day interaction for erythrocyte count, hemoglobin concentration, and hematocrit percentage, and on day 40, the values of these variables were higher in the blood of piglets in groups AO+YW-1 and AO+YW-2 when compared to NC (*p* = 0.01). Also on day 40, a lower platelet count was observed in the blood of piglets in groups AO+YW-1 and AO+YW-2 compared to NC (*p* = 0.01). On day 14, a higher neutrophil count was observed in the blood of piglets in AO+YW-2 compared to the other two groups (*p* = 0.05). There was no treatment effect or treatment × day interaction for total leukocyte count, lymphocytes, eosinophils and monocytes (*p* > 0.05).

### 3.4. Serum Biochemistry

Total protein, albumin and globulin results are presented in Table 5. There was no treatment effect or treatment × day interaction for total protein and albumin levels (*p* > 0.05). Lower globulin levels were observed in the serum of piglets in the AO+YW-2 group when compared to the other two groups on day 40 (*p* = 0.05).

### 3.5. Short-Chain Fatty Acid Profile in Feces

The results of the short-chain fatty acid (SCFA) profile in the piglets’ feces are presented in Table 6. On day 40, a higher amount of total SCFA was observed in the feces of AO+YW-2 piglets compared to the other groups (*p* = 0.01). There was no difference between groups for the amount of acetic acid in the feces (*p* > 0.05). On day 14, a lower level of propionic acid was observed in the feces of piglets in the AO+YW-1 group when compared to NC (*p* = 0.05); on day 40, the lower amount of propionic acid remained lower in piglets in the AO+YW-1 group when compared to AO+YW-2. The levels of butyric acid in the feces were higher in the feces of piglets in the AO+YW-2 group when compared to the other two groups on day 40 (*p* = 0.01). On day 40, higher levels of isovaleric acid in feces were observed in the AO+YW-1 group compared to NC (*p* = 0.02). Regarding valeric acid levels, on day 14, a lower amount of this acid was observed in the feces of piglets in the AO+YW-1 and AO+YW-2 groups when compared to NC (*p* = 0.02).

### 3.6. Immunological Response

The results of immunological response biomarkers are presented in Table 7. Lower concentrations of C-reactive protein were observed in the serum of piglets in the AO+YW-1 and AO+YW-1 groups when compared to the NC group on days 14 and 40 (*p* = 0.05). On day 40, lower ferritin levels were also reported in piglets in the AO+YW-2 group when compared to the NC group (*p* = 0.02). Lower serum concentrations of TNF-α in piglets in the AO+YW-1 and AO+YW-2 groups when compared to the NC group on day 40 were observed (*p* = 0.01). Also, at the end of the experiment (d40), higher concentrations of IL-10 were observed in the serum of both the AO+YW-1 and AO+YW-2 groups when compared to the NC group (*p* = 0.01).

## 4. Discussion

Piglets that consumed a combination of organic acids and yeast wall at the lowest dose (AO+YW-1) showed the highest WG and feed intake between days 1 and 26, showing that the combination of additives had a performance-enhancing effect on piglets in the nursery phase. The highest dose of the additive (AO+YW-2) also had an anti-inflammatory response but was not as efficient as the others when we observed daily WG; furthermore, between days 1 and 14, the piglets had worse feed conversion. Considering the total experimental period, there was no effect of the treatment on feed conversion, unlike another study that used organic acids and consequently improved feed conversion [5]. In general, the dose of 2 kg/t slightly impaired piglet performance, which resulted in poorer feed conversion in the first 14 days of nursery; even though no serum inflammatory process was observed, this does not prevent local inflammation in the intestine from occurring, which interfered with absorption. Furthermore, organic acids act by reducing the pH of the feed in the gastrointestinal tract, thus altering the growth conditions of microorganisms and directly inhibiting the growth of specific bacteria [6,13], which deserves to be investigated in future studies by our research group.

This study’s success in growth performance was due to the combination of organic acids and prebiotics, both already used in animal feed as an additive. Recent studies reported that, for organic acids [5,6], beta-glucans + MOS, when used in the diet of weaning pigs, are potentially beneficial to growth performance on days 1–21, bacterial population balance in feces and diarrhea incidence reduction [14]. Another study showed that the consumption of beta-glucans could attenuate intestinal damage in weaned pigs upon enterotoxigenic *Escherichia coli* challenge, related to the suppressed secretion of inflammatory cytokines and enhanced serum immunoglobulins, as well as improved intestinal epithelium functions and microbiota [15,16]. Piglets in the AO+YW-1 group (organic acids and prebiotic) had a smaller number of animals to be medicated, medication only for diarrhea, in the higher dose of the additive (AO+YW-2). The number of animals medicated was double that of the AO+YW-1 group, which had piglets treated via injection for encephalitis and cough, in addition to cases of diarrhea. This shows that, despite the higher dose of the additive, there were no benefits in preventing antibiotic treatment, which may have been reflected in the worse feed conversion of these animals of AO+YW-2.

Piglets that consumed the combination of organic acids and prebiotics (AO+YW-1 and AO+YW-2 groups) had higher erythrocyte counts, hemoglobin concentration, and hematocrit percentage at the end of the nursery compared negative control, indicating benefits of the additive since these biomarkers remained within the reference values [17], and a more significant number of erythrocytes can contribute to tissue oxygenation and, thus, indirectly contribute to the better growth performance of piglets. Since the 1980s, there have been records that glucans can affect the bone marrow, stimulating hematopoiesis [18], which explains our effect on the erythrogram. We cannot rule out the simultaneous action of organic acids in this result, primarily since it is known, for example, that acetate supplementation augments stress erythropoiesis in an acetate-dependent acetyl CoA synthetase in two manners, as well as in acquired and inherited chronic anemia mouse models, acetate supplementation increases hormone erythropoietin expression and the resting hematocrit [19].

The lower neutrophil count in the blood of piglets in the AO+YW-1 group is probably related to lower cases of health problems in piglets (enteric, respiratory, and systemic) and lower number of medicated animals. The lack of effects of the combination of organic acids and prebiotics (1 or 2 kg/ton) on leukocytes was not expected since it is well known that beta-glucan acts as an immunostimulant to activate immune cells by binding to its specific receptor dectin-1, a c-type lectin receptor expressed on the surface of macrophages [20,21], and the consumption of organic acids is capable of modulating the immune response [22]. Therefore, these same mechanisms can explain our results.

We understand that since there was no stimulation of white blood cells, there was no greater production of total proteins and globulins despite the existence of immunological and inflammatory pathways being influenced by the consumption of additives by the piglets. This is because C-reactive proteins and ferritin, two positive acute-phase proteins, were at lower levels in the serum of piglets that consumed the highest dose of the additive (AO+YW-2). Knowing that these proteins are associated with inflammation and inflammatory processes, the hypothesis arose that the additive would have an anti-inflammatory effect. This hypothesis was confirmed when we measured the levels of TNF-α and IL-10 because TNF-α is a pro-inflammatory cytokine (reduced in the serum of piglets). At the same time, IL-10 is an anti-inflammatory cytokine (increased in the serum of piglets) that helps regulate the immune response and limit excessive inflammation [23,24]. These cytokines, like others, play an essential role in different aspects of the body’s immune response and homeostasis [25]. According to the literature, prebiotics and organic acids aid in the production of lactic acid and acetic acid and so maintain a stable intestinal pH, favoring the activity of digestive enzymes, which contributes to the maintenance of intestinal health, with a reduction in intestinal inflammation due to the lower expression of inflammatory cytokines [26]. Therefore, we understand that the combination of organic acids and yeast cell walls can act as an anti-inflammatory additive, which is desirable in production animals, which reduced the use of ATP to produce inflammation and began to use it to improve performance.

The combination AO+YW-2 increased the levels of SCFA in feces at the end of the nursery phase but also altered the levels of propionic acid, butyric acid, isovaleric acid, and valeric acid in feces between the groups, unlike acetic acid, which, despite being one of the organic acids used as an additive, did not affect it in feces. According to the literature, the profile of SCFA in feces indicates bacterial activity and the metabolic state of the piglets’ digestive tract, influenced by the diet and intestinal health conditions of the animals [27]. SCFA can provide piglet energy, give resistance to pathogenic microorganisms, and maintain the intestinal health of animals [27,28,29]. In addition, it is known that SCFAs are absorbed by the epithelial cells of the colon and used as energy, reducing the incidence of infectious diseases [28]. Researchers have previously demonstrated that organic acids increase total volatile fatty acid content in piglet feces [27,29]. According to the literature, the yeast cell wall compounds, even at low dietary concentrations, affect fecal SCFA production, reduce the fecal pH, and modulate the fecal microbiota [30]. Therefore, the combination of prebiotic and organic acid participates in the changes in volatile fatty acids in the feces of piglets.

The results of our research show a strong relationship between the improvement in the immune response and anti-inflammatory response in piglets that consumed additives and the frequency of occurrence of infectious diseases, with emphasis on the lower dose of the additive, where no cases of encephalitis were observed. According to the literature, encephalitis in animals is an opportunistic infection, resulting from immunosuppression or simply a lower immune response [31,32]. In addition, the exacerbated inflammatory process leads to a condition of oxidative stress, which makes the animals more vulnerable to infectious diseases [33]. Therefore, this justifies why infectious diseases were more common in control piglets compared to AO+YW-1.

In summary, the additive blend used here at a 1 kg/ton dose increased WG in the initial nursery phase, having an anti-inflammatory effect and stimulating erythropoiesis. These data are exciting and desirable, but in addition to this, the number of sick animals was lower, as was the number of injectable drug interventions, compared to the NC group, which resulted in lower production costs with medication expenditure and, mainly, the animals stopping eating and gaining weight. The combination of this additive was established to act in stimulating the immune response of the intestinal tract and modulating the microbiota, expecting an increase in microorganisms beneficial to intestinal health. The dose of 2 kg/ton (AO+YW-2) had positive results for animal health, but it ended up not being an interesting dose because it did not enhance weight gain, worsened feed conversion, and the number of animals treated was greater when compared to the AO+YW-1 group.

## 5. Conclusions

The combination of organic acids and yeast wall used at a dose of 1 kg/ton proved to be an option additive for the diet of piglets in the nursery phase to enhance weight gain and reduce the number of medicated animals. The additive combination stimulated the production of erythrocytes, which can improve tissue oxygenation and favored the WG of piglets in the AO+YW-1 group. Increased IL-10 levels combined with reduced TNF-α levels in the blood of piglets indicate the additive’s anti-inflammatory effect in the AO+YW-1 and AO+YW-2 groups. The use of the additive at a dose of 1 kg/ton reduced the number of sick animals and drug interventions. Finally, ingesting the combination of organic acids and prebiotics changed the fatty acid profile in the feces of the piglets, emphasizing the higher concentration of SCFAs in piglets that consumed 2 kg per ton.

## Figures and Tables

**Table 1:** Ingredients and calculated composition of diets used in piglet feed.

Experimental Period	d1–7	d8–14	d15–25	26–40
Ingredients, g/kg	Pre-Initial I	Pre-Initial II	Initial I	Initial II
Ground corn	180.08	371.55	512.45	592.76
Pre-gelatinized corn	200.00	115.00	50.00	-
Soybean meal	194.90	220.00	218.20	217.94
Micronized soybean	80.00	88.54	125.00	142.00
Whey powder	200.00	100.00	50.00	-
Egg flour	40.00	20.00	-	-
Soybean Protein concentrate	30.00	15.00	-	-
Sugar	30.00	25.00	-	-
Calcitic limestone	10.10	7.30	6.82	6.77
Salt	-	2.50	3.95	4.40
Dicalcium phosphate	7.90	9.75	11.75	13.20
Sodium bicarbonate	4.66	3.10	3.30	4.50
Basemix ^1^	6.00	6.00	6.00	6.00
L-Lysine—HCl	3.86	3.95	4.00	4.03
DL-Methionine	2.38	2.70	1.78	1.73
L-Threonine	3.90	3.92	3.40	3.43
L-Tryptophan	0.24	0.24	0.27	0.26
L-Isoleucine	0.97	0.13	-	-
L-Valine	0.16	0.97	0.73	0.63
Carbohydrases	0.10	0.10	0.10	0.10
Phytase	0.05	0.05	0.05	0.05
Flavoring agent	0.20	0.20	0.20	0.20
Antioxidant additive	1.00	1.00	1.00	1.00
Zinc oxid 75% Zn	3.50	3.00	1.00	1.00
Calculated comsposition ^2^				
Calcium (Ca), %	0.84	0.70	0.70	0.70
Phosphorus availability, %	0.38	0.35	0.35	0.35
Sodium (Na), %	0.34	0.30	0.30	0.30
Chlorine (Cl), %	0.30	0.33	0.35	0.32
Metabolizable energy (ME), kcal/kg	3442	3374	3344	3345
Digestible protein, %	22.0	21.3	20.0	20.2
Digestible lysine, %	1.45	1.34	1.28	1.28
Digestible methionine, %	0.56	0.56	0.45	0.44
Digestible methionine + Cysteine, %	0.87	0.86	0.73	0.73
Digestible threonine, %	1.15	1.08	0.99	0.99
Digestible tryptophan, %	0.27	0.25	0.24	0.24
Digestible isoleucine, %	0.95	0.83	0.78	0.78
Digestible valine, %	0.97	0.98	0.92	0.92
Zinc, ppm	2670	2287	783	778

^1^ Minimum levels per kg of feed on an as-fed basis: Pre-initial I—Cu 150.45 mg, Fe 100 mg, I 1.8 mg, Mn 57.75 mg, Se 0.41 mg, Zn 2750.16 mg, Vitamin D 12,150 IU, Vitamin E 2430 IU, Vitamin E 102 IU, Vitamin K3 4.05 mg, B1 1.89 mg, B2 5.4 mg, B6 2.03 mg, B12 24.3 mg, Folic Acid 0.54 mg, Pantothenic Acid 22.06 mg, Nicotinic Acid 41.82 mg, Biotin 0.14 mg, Cholin 1159.21 mg, Phytase 500 FTU/kg; Pre-initial II—Cu 150.45 mg, Fe 100 mg, I 1.8 mg, Mn 57.75 mg, Se 0.41 mg, Zn 2375.16 mg, Vitamin D 12,150 IU, Vitamin E 2430 IU, Vitamin E 102 IU, Vitamin K3 4.05 mg, B1 1.89 mg, B2 5.4 mg, B6 2.03 mg, B12 24.3 mg, Folic Acid 0.54 mg, Pantothenic Acid 22.06 mg, Nicotinic Acid 41.82 mg, Biotin 0.14 mg, Cholin 1292.79 mg, Phytase 500 FTU/kg; Initial I—Cu 150.45 mg, Fe 100 mg, I 1.8 mg, Mn 57.75 mg, Se 0.41 mg, Zn 875.16 mg, Vitamin D 12,150 IU, Vitamin E 2430 IU, Vitamin E 62 IU, Vitamin K3 4.05 mg, B1 1.89 mg, B2 5.4 mg, B6 2.03 mg, B12 24.3 mg, Folic Acid 0.54 mg, Pantothenic Acid 22.06 mg, Nicotinic Acid 41.82 mg, Biotin 0.14 mg, Cholin 1390.09 mg, Phytase 500 FTU/kg; Initial II—Cu 150.45 mg, Fe 100 mg, I 1.8 mg, Mn 57.75 mg, Se 0.41 mg, Zn 125.16 mg, Vitamin D 12,150 IU, Vitamin E 2430 IU, Vitamin E 62 IU, Vitamin K3 4.05 mg, B1 1.89 mg, B2 5.4 mg, B6 2.03 mg, B12 24.3 mg, Folic Acid 0.54 mg, Pantothenic Acid 22.06 mg, Nicotinic Acid 41.82 mg, Biotin 0.14 mg, Cholin 1435.76 mg, Phytase 500 FTU/kg. ^2^ Values calculated based on the nutritional composition proposed by Rostagno et al. [12].

**Table 2:** Recording of clinical signs/pathologies in piglets in the nursery phase and drugs used.

Groups	Clinical Sign/Pathology	Animals Number and Percentage	Drugs	Doses Number (Mean)
Negative control—NC	Diarrhea	6 (22.2%)	Lincomycin	4.16
	Encephalitis	5 (18.5%)	Amoxicillin + colistin	3.60
	Persistent cough	3 (11.1%)	Amoxicillin + colistin	3.0
AO+YW-1	Diarrhea	4 (14.8%)	Lincomycin	3.0
AO+YW-2	Diarrhea	8 (29.6%)	Lincomycin	3.75
	Encephalitis	1 (3.70%)	Amoxicillin + colistin	5.0
	Persistent cough	2 (7.40%)	Amoxicillin + colistin	3.0

**Table 3:** Body weight, weight gain, and feed conversion of nursery piglets fed a combination of organic acids and yeast wall.

Variables	NC	AO+YW-1	AO+YW-2	SEM	P: Trat.	P: Trat. × Day
Body weight, kg						
d1	7.17	7.17	7.17	0.110	0.08	0.05
d7	7.39	7.48	7.35	0.116		
d14	9.57	10.1	9.66	0.111		
d26	16.2	16.6	16.1	0.122		
d40	24.1 ^b^	25.4 ^a^	24.6 ^ab^	0.123		
Average daily WG, kg/day						
d1–7	0.032	0.045	0.026	0.01	0.74	-
d1–14	0.172 ^b^	0.211 ^a^	0.178 ^b^	0.01	0.01	-
d1–26	0.347 ^b^	0.362 ^a^	0.344 ^b^	0.02	0.01	-
d1–40	0.424 ^b^	0.456 ^a^	0.435 ^b^	0.03	0.01	-
Average daily feed consumption, kg/day						
d1–7	0.142	0.163	0.148	0.01	0.43	-
d1–14	0.275	0.296	0.277	0.01	0.16	-
d1–26	0.493 ^b^	0.519 ^a^	0.501 ^b^	0.02	0.02	-
d1–40	0.677 ^a^	0.678 ^a^	0.665 ^b^	0.03	0.05	-
Feed conversion, kg/kg						
d1–7	4.43	3.62	5.69	0.85	0.64	-
d1–14	1.45 ^b^	1.45 ^b^	1.67 ^a^	0.11	0.01	-
d1–26	1.43	1.44	1.46	0.05	0.79	-
d1–40	1.54	1.49	1.53	0.05	0.29	-

The treatments were negative control (NC: no promoter), and a combination of organic acids and yeast cell wall (AO+YW-1 and AO+YW-2 which refers to 1 and 2 kg/ton, respectively). Note: Within a line, differ when *p* ≤ 0.05, illustrated by different letters (a,b).

**Table 4:** Hematologic variables of nursery piglets fed a combination of organic acids and yeast wall.

Variables	NC	AO+YW-1	AO+YW-2	SEM	P: Treat.	P: Treat. × Day
Erythrocytes (×10^6^ cell/μL)					0.37	0.01
D14	6.76	6.60	6.58	0.03		
D40	6.46 ^b^	7.87 ^a^	8.07 ^a^	0.03		
Hemoglobin (g/dL)					0.44	0.01
D14	10.8	10.4	10.4	0.34		
D40	10.4 ^b^	12.7 ^a^	13.1 ^a^	0.37		
Hematocrit (%)					0.40	0.01
D14	38.8	37.2	37.5	1.40		
D40	36.8 ^b^	44.2 ^a^	45.4 ^a^	1.42		
Platelets (×10^3^/μL)					0.11	0.01
D14	479	427	417	13.60		
D40	456 ^a^	395 ^b^	398 ^b^	7.78		
Leukocytes (×10^3^ cell/μL)					0.21	0.14
D14	10.3	9.59	11.0	0.58		
D40	8.97	8.82	8.42	0.52		
Neutrophil (×10^3^ cell/μL)					0.03	0.05
D14	4.87 ^ab^	4.20 ^b^	5.90 ^a^	0.49		
D40	3.96	3.40	3.80	0.51		
Lymphocytes (×10^3^ cell/μL)					0.55	0.12
D14	5.27	5.27	4.89	0.43		
D40	4.93	5.27	4.47	0.40		
Eosinophil (×10^3^ cell/μL)					0.86	0.94
D14	0.04	0.07	0.05	0.10		
D40	0.06	0.09	0.07	0.10		
Monocyte (×10^3^ cell/μL)					0.71	0.87
D14	0.09	0.08	0.12	0.14		
D40	0.09	0.08	0.14	0.13		

The treatments were negative control (NC: no promoter), and a combination of organic acids and yeast cell wall (AO+YW-1 and AO+YW-2 which refers to 1 and 2 kg/ton, respectively). Note: Within a line, differ when *p* ≤ 0.05, illustrated by different letters (a,b).

**Table 5:** Total protein, albumin, and globulin of nursery piglets fed a combination of organic acids and yeast wall.

Variables	NC	AO+YW-1	AO+YW-2	SEM	P: Trat.	P: Trat. × Day
Total protein (g/dL)					0.31	0.18
D14	4.62	5.01	4.62	0.23		
D40	5.01	5.09	4.28	0.21		
Albumin (g/dL)					0.49	0.26
D14	2.22	2.28	2.27	0.12		
D40	2.19	2.49	2.31	0.11		
Globulin (g/dL)					0.05	0.05
D14	2.48 ^a^	2.44 ^a^	2.16 ^b^	0.12		
D40	2.74 ^a^	2.86 ^a^	2.09 ^b^	0.13		

The treatments were negative control (NC: no promoter), and a combination of organic acids and yeast cell wall (AO+YW-1 and AO+YW-2 which refers to 1 and 2 kg/ton, respectively). ^a,b^ Within a line, different (*p* ≤ 0.05) is illustrated by different letters.

**Table 6:** Short-chain fatty acid profile in the feces of nursery piglets fed a combination of organic acids and yeast wall.

Fatty Acid	NC	AO+YW-1	AO+YW-2	SEM	P: Trat.	P: Trat. × Day
Acetic, mmol/kg					0.82	0.37
d14	72.5	77.9	73.8	6.08		
d40	92.7	84.3	93.4	6.12		
propionic, mmol/kg					0.22	0.05
d14	25.2 ^a^	19.5 ^b^	22.6 ^ab^	0.41		
d40	40.3 ^ab^	36.3 ^b^	44.4 ^a^	0.40		
Butyric, mmol/kg					0.36	0.01
d14	19.7	16.6	17.5	0.38		
d40	19.1 ^b^	22.3 ^b^	28.1 ^a^	0.42		
Isovaleric, mmol/kg					0.10	0.02
d14	3.27	2.52	2.62	0.07		
d40	3.59 ^b^	5.46 ^a^	4.46 ^ab^	0.07		
Valeric, mmol/kg					0.21	0.02
d14	7.30 ^a^	4.39 ^b^	5.44 ^b^	0.35		
d40	9.26	10.6	11.6	0.39		
Total SCFA, mmol/kg					0.15	0.01
d14	128	121	122	1.85		
d40	165 ^b^	159 ^b^	182 ^a^	2.40		

The treatments were negative control (NC: no promoter), and a combination of organic acids and yeast cell wall (AO+YW-1 and AO+YW-2 which refers to 1 and 2 kg/ton, respectively). Note Within a line, differ when *p* ≤ 0.05, illustrated by different letters (a,b).

**Table 7:** C-reactive protein, ferritin, and cytokines in the serum of nursery piglets fed a combination of organic acids and yeast wall.

Variables	NC	AO+YW-1	AO+YW-2	SEM	P: Trat.	P: Trat. × Day
C-reactive protein, g/dL					0.01	0.05
d14	0.39 ^a^	0.29 ^ab^	0.25 ^b^	0.07		
d40	0.38 ^a^	0.26 ^b^	0.23 ^b^	0.09		
Ferritin, g/dL					0.22	0.02
d14	0.28	0.26	0.25	0.02		
d40	0.24 ^a^	0.20 ^ab^	0.17 ^b^	0.02		
TNF-α, pg/mL					0.28	0.01
d14	251	238	245	3.32		
d40	227 ^a^	197 ^b^	152 ^c^	3.85		
IL-10, pg/mL					0.05	0.01
d14	366	372	378	7.10		
d40	247 ^c^	342 ^b^	408 ^a^	7.09		

The treatments were negative control (NC: no promoter), and a combination of organic acids and yeast cell wall (AO+YW-1 and AO+YW-2 which refers to 1 and 2 kg/ton, respectively). ^a–c^ Within a line, differ when *p* ≤ 0.05, illustrated by different letters.

## Data Availability

All authors approved the manuscript for publication. This manuscript article includes all the data generated or analyzed during this study.

## Supplementary Materials

The following supporting information can be downloaded at https://www.mdpi.com/article/10.3390/ani15071051/s1, Table S1: Standardization of quantification of short-chain fatty acids in feces.
