# Supplementary material for: Effects on Performance, Immunological Response and Short-Chain Fatty Acid Profile in Feces of Nursery Piglets Fed with Organic Acids and Yeast Wall
Source: Animals (Basel). 2025 Apr 4;15(7):1051. doi: 10.3390/ani15071051 (PMC11988164; doi:10.3390/ani15071051)
Supplement: Supplementary file 1 [file animals-15-01051-s001.zip › animals-3420963-supplementary.pdf]

Table S1. Standardization of quantification of short-chain fatty acids in feces.

Item	**Acetic acid**	**Propionic acid**	**Butyric acid**	**Isovaleric acid**
R^2	0.9995	0.9998	0.9995	0.9995
Equation	y = 0.0115x + 0.0066	y = 0.0146x - 0.0034	y = 0.0300x + 0.0009	y = 0.0374x + 0.0004
Linear range (mmol L^{-1})*	1.07 - 68.87	0.46 - 58.45	0.33 - 42.43	0.14 - 18.53
LOD (mmol L^{-1})	0.54	0.46	0.33	0.14
LOQ (mmol L^{-1})	1.07	0.91	0.66	0.29
Accuracy	108.11	95.96	105.44	106.70
Repeatability (RSD)	1.05	2.91	0.89	1.55

* The linear range. LOD (limit of detection) and LOQ (limit of quantitation) were expressed in mmol L^{-1} of SCFA.
